# Utilization and costs of epidermal growth factor receptor mutation testing and targeted therapy in Medicare patients with metastatic lung adenocarcinoma

**DOI:** 10.1186/s12913-022-07857-y

**Published:** 2022-04-09

**Authors:** Chan Shen, Rolfy A. Perez Holguin, Eric Schaefer, Shouhao Zhou, Chandra P. Belani, Patrick C. Ma, Michael F. Reed

**Affiliations:** 1 Department of Surgery, College of Medicine, The Pennsylvania State University, Hershey, PA USA; 2 Department of Public Health Sciences, College of Medicine, The Pennsylvania State University, PA Hershey, USA; 3 Penn State Cancer Institute, Hershey, PA USA; 4 Department of Medicine, College of Medicine, The Pennsylvania State University, Hershey, PA USA

**Keywords:** EGFR mutation testing, Targeted therapy, SEER-Medicare database, Adenocarcinoma

## Abstract

**Background:**

Guidelines in 2013 and 2014 recommended Epidermal Growth Factor Receptor (EGFR) testing for metastatic lung adenocarcinoma patients as the efficacy of targeted therapies depends on the mutations. However, adherence to these guidelines and the corresponding costs have not been well-studied.

**Methods:**

We identified 2362 patients at least 65 years old newly diagnosed with metastatic lung adenocarcinoma from January 2013 to December 2015 using the SEER-Medicare database. We examined the utilization patterns of EGFR testing and targeted therapies including erlotinib and afatinib. We further examined the costs of both EGFR testing and targeted therapy in terms of Medicare costs and patient out-of-pocket (OOP) costs.

**Results:**

The EGFR testing rate increased from 38% in 2013 to 51% and 49% in 2014 and 2015 respectively. The testing rate was 54% among the 394 patients who received erlotinib, and 52% among the 42 patients who received afatinib. The median Medicare and OOP costs for testing were $1483 and $293. In contrast, the costs for targeted therapy were substantially higher with median 30-day costs at $6114 and $240 for erlotinib and $6239 and $471 for afatinib.

**Conclusion:**

This population-based study suggests that testing guidelines improved the use of EGFR testing, although there was still a large proportion of patients receiving targeted therapy without testing. The costs of targeted therapy were substantially higher than the testing costs, highlighting the need to improve adherence to testing guidelines in order to improve clinical outcomes while reducing the economic burden for both Medicare and patients.

**Supplementary Information:**

The online version contains supplementary material available at 10.1186/s12913-022-07857-y.

## Introduction

Chemotherapy has historically been the centerpiece of treatment for metastatic non-small cell lung cancer (NSCLC). This started to change in the early 2000s with the development of targeted therapies, specifically tyrosine kinase inhibitors (TKIs), which are used in patients with certain activating EGFR mutations [1]. In patients with EGFR sensitizing mutations, targeted therapy has superior clinical efficacy and progression-free survival compared to chemotherapy [2, 3]. The development of these therapies has led to changes in the guidelines for the treatment of NSCLC. EGFR mutation testing was first recommended by the American Society of Clinical Oncology (ASCO) in a provisional clinical opinion in 2011 [4]. Similarly, in 2012 the National Comprehensive Cancer Network (NCCN) recommended testing for EGFR mutations in all patients with lung adenocarcinoma [5]. As a result, the standard of care for patients with advanced staged NSCLC shifted toward treatment guided by molecular genotypes by 2013 [6]. In July 2013, the College of American Pathologists/International Association for the Study of Lung Cancer/Association for Molecular Pathology (CAP/IASLC/AMP) released guidelines recommending that EGFR mutation testing be used to guide the choice of EGFR inhibitor treatment [7]. These guidelines were endorsed by ASCO in October 2014 [8].

EGFR inhibitors currently commercially available in the U.S. include erlotinib, afatinib, gefitinib, dacomitinib, and osimertinib. Erlotinib and afatinib were approved as first-line treatment of metastatic NSCLC with EGFR mutations in 2013 [9, 10], followed by gefinitib in 2015 [11] and dacomitinib and osimertinib in 2018 [12, 13]. Multiple studies have shown that the efficacy of EGFR targeted therapy depends on the appropriate selection of patients with sensitizing mutations [14–17], which highlights the importance of molecular testing for proper patient selection.

Prior studies have demonstrated underutilization of EGFR molecular testing, as well as disparities in testing based on race, income, and geographical area. However, these studies only examined patients diagnosed before 2013 [18–20]. Since the molecular testing guidelines by CAP/IASLC/AMP were released in 2013 [7], and then endorsed by ASCO in 2014 [8], data prior to 2013 does not reflect adherence to these newer guidelines. A study[21] using the MarketScan database analyzed utilization patterns in testing for EGFR mutations from 2013 to 2014; however, it did not include histology information and therefore was unable to exclude patients with lung cancer histologies with lower rate of EGFR mutations [1, 21].

Another important knowledge gap is the lack of studies addressing the cost of EGFR testing. Numerous reports have shown the substantial economic burden of targeted therapies on both patients and Medicare [22–26]. However, they did not examine the cost burden of EGFR testing.

The current study is a population-based analysis using the SEER-Medicare database to examine patterns of testing for EGFR mutations and identify factors associated with testing in the Medicare population. Further, this study aims to evaluate the cost of EGFR testing in the context of EGFR targeted therapy.

## Method

### Data source

The data source used in the study was the Surveillance, Epidemiology, and End Results (SEER) registry data linked with Medicare claims from the National Cancer Institute (NCI). It is available at: https://healthcaredelivery.cancer.gov/seermedicare/. The SEER registries cover approximately 28% of the U.S. population [27]. The SEER cancer registry data provide information on both patients’ demographics and clinical characteristics such as tumor stage and histology. The linkage to Medicare claims data enriches the data by adding information on the health care utilization of the patients both before and after cancer diagnosis.

### Study cohort

We identified patients at least 65 years old newly diagnosed with metastatic lung adenocarcinoma from January 2013 to December 2015 from the SEER-Medicare database. Patients had metastatic lung cancer as their first primary cancer and had International Classification of Diseases for Oncology, 3rd Edition (ICD-O-3) code 8140 indicating lung adenocarcinoma histology. We required that the patients survived at least 6 months after diagnosis, and had continuous enrollment in Medicare Parts A, B, D and no health maintenance organization (HMO) coverage during the 6 months prior to diagnosis through 6 months after diagnosis to ensure complete claims information to capture pre-existing comorbidities and the utilization of EGFR testing and EGFR targeted therapy. Detailed inclusion and exclusion steps are provided in Additional file 1: Appendix Fig. 1.

### Utilization of EGFR testing and EGFR targeted therapy

We identified EGFR testing based on Current Procedural Terminology (CPT) code 81,235. The utilization of EGFR targeted therapies was determined using the following National Drug Codes (NDCs): 50,242–0062-01, 50,242–0063-01, 50,242–0064-01, 54,868–5290-00, 54,868–5447-00, 54,868–5474-00 for erlotinib and 00,597–0137-30, 00,597–0137-90, 00,597–0138-30, 00,597–0138-95, 00,597–0141-30 for afatinib. We also explored the use of gefitinib using the following NDCs: 00,310–0482-30, 00,310–0482-93. However, we only found two patients in our sample with gefinitib prescriptions, likely because gefitinib was only approved in late 2015 at the end of our study cohort inclusion. Therefore, we did not include gefitinib in our final analysis.

Given the possibility that EGFR testing could be performed as part of a panel test and laboratory developed test of cell free DNA we performed a sensitivity analysis including the CPT codes: 81,445 and 81,455 (Genomic Sequencing Procedures and Other Molecular Multianalyte Assays) and 81,479 (unlisted molecular pathology procedure). Of note, any EGFR testing performed as part of a research protocol may not be captured in this analysis.

### Costs of EGFR testing and EGFR targeted therapy

We examined the costs of EGFR testing and EGFR targeted therapy from both the payer’s perspective (i.e. Medicare) and the patient’s perspective. Specifically, we examined costs from payer’s perspective based on Medicare payment amount while we examined costs from patient’s perspective based on patient out-of-pocket (OOP) cost documented in the Medicare claims. For the EGFR testing cost, we calculated the total amount paid by Medicare and by the patient respectively for the visit involving EGFR testing. For the EGFR targeted therapy cost, we studied the average Medicare payment amount for a monthly (30-day) prescription. All costs were inflated to 2016 dollars using the medical care component of the consumer price index [28].

### Patient characteristics

The demographic and socioeconomic characteristics included in this study were age, sex (male, female), race (White, Black, Asian, other), ethnicity (Hispanic, non-Hispanic), marital status (married, other), urban/rural status (big metro, metro, urban, less urban/rural), census tract poverty prevalence (0 to < 5%, 5% to < 10%, 10% to < 20%, 20% to 100%), and Medicaid dual eligibility (yes, no). We calculated the Quan modification of the Charlson Comorbidity Index [29] (CCI) based on all claims that occurred within 6 months prior to diagnosis via International Classification of Diseases 9th Revision (ICD-9) and 10^th^ revision (ICD-10) diagnosis codes. We also included the year of diagnosis and an indicator for the use of radiation therapy within 6 months of diagnosis based on ICD-9 and ICD-10 diagnosis and procedure codes, CPT codes and revenue center codes from the Medicare claims.

### Statistical analyses

We investigated use of an EGFR test within 6 months of diagnosis by patient characteristics. We used chi-squared tests for categorical variables and Wilcoxon tests for continuous variables to test for differences in EGFR use by patient characteristics. A multivariable logistic regression model was used to further examine the association between patient characteristics and use of EGFR testing. Age and month of diagnosis were modeled as linear effects, which was appropriate based on spline fits and graphical analyses. All other variables were categorical and used reference values. We reported odds ratios (ORs) and corresponding 95% confidence intervals (CIs) for the parameters in the logistic model. We provided the mean, standard deviation, median, interquartile range, and range and histograms for the costs of EGFR testing and targeted therapy.

This manuscript was written according to the STROBE guidelines, and the checklist is provided as Supplementary Material [30]. The statistical analyses were conducted in SAS 9.4 (SAS Institute, Cary, NC) and R 3.6.0 (R Core Team, Vienna, Austria). The Institutional Review Board approved this retrospective observational study, and all patients in the database had been de-identified.

## Results

A total of 2,362 newly diagnosed metastatic lung adenocarcinoma patients met all inclusion/exclusion criteria and were included in the study. Among the sample, 1,086 (46%) received EGFR testing within 6 months of diagnosis. To ensure that the 6 month time window to capture EGFR testing was sufficient, we also evaluated the time from cancer diagnosis to EGFR testing, and found that close to half (46.7%) of these patients received the testing during the month of diagnosis, 34.6% received it in the month after diagnosis, and only 1.4% received the testing in the 6^th^ month after diagnosis.

Table 1 shows the characteristics of the study cohort stratified by whether EGFR testing was performed. EGFR testing significantly increased from year 2013 to 2014 (38% to 51%). In the study cohort, 394 patients (16.7%) received erlotinib and 42 (1.8%) received afatinib. For patients treated with either therapy, close to half did not receive EGFR testing (45.7% for erlotinib, and 47.6% for afatinib). We also found significant differences in the use of EGFR testing by race, marital status, urban/rural status, census tract poverty rate, Medicaid dual eligibility, and Charlson comorbidity score.

**Table 1:** Patient characteristics stratified by EGFR testing

	**No EGFR test** **(*N* = 1276)**	**EGFR** **Test** **(*N* = 1086)**	***p*-value**
**Erlotinib use**			< 0.001
No	1096 (85.9%)	872 (80.3%)	
Yes	180 (14.1%)	214 (19.7%)	
**Afatinib use**			0.401
No	1256 (98.4%)	1064 (98.0%)	
Yes	20 (1.6%)	22 (2.0%)	
**Year of diagnosis**			< 0.001
2013	496 (62.1%)	303 (37.9%)	
2014	379 (48.9%)	396 (51.1%)	
2015	401 (50.9%)	387 (49.1%)	
**Age at diagnosis**			0.738
66–69	340 (55.9%)	268 (44.1%)	
70–74	353 (53.7%)	304 (46.3%)	
75–79	303 (53.3%)	265 (46.7%)	
≥ 80	280 (52.9%)	249 (47.1%)	
**Sex**			0.457
Male	515 (55.0%)	422 (45.0%)	
Female	761 (53.4%)	664 (46.6%)	
**Race**			0.023
White	1003 (53.0%)	891 (47.0%)	
Black	128 (63.4%)	74 (36.6%)	
Asian	73 (51.0%)	70 (49.0%)	
Other (including unknown)	72 (58.5%)	51 (41.5%)	
**Ethnicity**			0.159
Non-Hispanic	1198 (53.7%)	1034 (46.3%)	
Hispanic	78 (60.0%)	52 (40.0%)	
**Marital status**			0.017
Not married/unknown	648 (56.5%)	498 (43.5%)	
Married	628 (51.6%)	588 (48.4%)	
**Urban/rural code**			0.024
Big metro	686 (52.2%)	628 (47.8%)	
Metro	362 (53.7%)	312 (46.3%)	
Urban	80 (58.8%)	56 (41.2%)	
Less urban/rural	148 (62.2%)	90 (37.8%)	
**Census tract poverty indicator**			0.003
0 to < 5%	296 (50.9%)	285 (49.1%)	
5% to < 10%	305 (49.9%)	306 (50.1%)	
10% to < 20%	365 (55.5%)	293 (44.5%)	
20% to 100%	309 (60.5%)	202 (39.5%)	
**Medicaid dual-eligible**			< 0.001
No	939 (52.0%)	866 (48.0%)	
Yes	337 (60.5%)	220 (39.5%)	
**Charlson score**			0.005
0	327 (49.7%)	331 (50.3%)	
1	422 (54.1%)	358 (45.9%)	
2	191 (52.8%)	171 (47.2%)	
≥ 3	336 (59.8%)	226 (40.2%)	
**Radiation**			0.636
No	634 (54.5%)	529 (45.5%)	
Yes	642 (53.5%)	557 (46.4%)	

Odds ratios from the multivariable logistic regression model for EGFR testing are shown in Table 2. The estimates indicate a significant increase in the uptake of EGFR testing over time with every one-month increase in calendar time being associated with 19% higher odds of receiving EGFR testing (OR = 1.19, 95%CI = [1.08–1.31], *p*-value < 0.001). Living in less urban or rural areas was associated with a 33% lower odds (OR = 0.67, 95%CI = [0.50,0.91], *p*-value = 0.01) of receiving EGFR testing compared to living in big metropolitan areas. A Charlson comorbidity score ≥ 3 was associated with lower odds of receiving testing (OR = 0.70, 95%CI = [0.56,0.89], *p*-value = 0.003) relative to patients with a zero comorbidity score.

**Table 2:** Results from the multivariable logistic regression model for EGFR testing

	**OR (95% CI)**	***p*-value**
Month of diagnosis, 1-month increase	1.19 (1.08–1.31)	< 0.001
Age at diagnosis, 5-year increase	1.02 (0.95–1.09)	0.59
Sex		
Female	1.06 (0.89–1.27)	0.49
Male (ref)	1	
Race		
White (ref)	1	
Black	0.76 (0.55–1.06)	0.11
Asian	1.15 (0.79–1.68)	0.47
Other (including unknown)	0.81 (0.55–1.20)	0.29
Ethnicity		
Non-Hispanic (ref)	1	
Hispanic	0.93 (0.62–1.37)	0.70
Marital status		
Not married/unknown (ref)	1	
Married	1.15 (0.97–1.38)	0.12
Urban/rural code		
Big metro (ref)	1	
Metro	0.93 (0.77–1.13)	0.47
Urban	0.79 (0.55–1.14)	0.21
Less urban/rural	0.67 (0.50–0.91)	0.010
Census tract poverty indicator		
0 to < 5% (ref)	1	
5% to < 10%	1.07 (0.85–1.34)	0.57
10% to < 20%	0.96 (0.76–1.20)	0.70
20% to 100%	0.90 (0.69–1.18)	0.44
Medicaid dual-eligibility		
Yes	0.81 (0.64–1.02)	0.07
No (ref)	1	
Charlson score		
0 (ref)	1	
1	0.87 (0.70–1.07)	0.18
2	0.92 (0.71–1.20)	0.55
≥ 3	0.70 (0.56–0.89)	0.003
Radiation		
Yes	0.99 (0.84–1.18)	0.95
No (ref)	1	

Table 3 describes the costs to Medicare and patient OOP costs of EGFR testing, as well as 30-day erlotinib and afatinib prescriptions. Large variations in both costs to Medicare and patient OOP costs were observed. Medicare carried most of the cost burden of both EGFR testing and targeted therapy prescriptions. The costs of EGFR targeted therapies were substantially higher than the EGFR testing. Specifically, the median cost of EGFR testing to Medicare was $1,483 with an interquartile range (IQR) of ($798, $2,234), while the median patient OOP cost was $293 with an IQR of ($96, $477). The median Medicare costs for 30 days of erlotinib and afatinib prescriptions were $6,114 (IQR: $5,460, $6,615) and $6,239 (IQR: $5,898, $6,841) respectively, while the corresponding median OOP costs were $240 (IQR: $1, $973) and $471 (IQR: $1, $902), respectively. Considering that the vast majority (> 95%) of these prescriptions were for 30 days, and the median number of prescriptions filled by patients using erlotinib and afatinib were five and four respectively during the 6 months period, the total costs of EGFR targeted therapies during this time period were substantial. Further, we found no statistical difference in the number of prescriptions between patients who received EGFR testing versus those who did not. Detailed descriptive statistics on the number of prescriptions are presented in Table 4. Of note, the cost of an office visit was included in our cost analysis for EGFR testing. However, in some practices an additional visit would not be required for EGFR testing. In this case, the cost of testing would be lower than what we have reported.

**Table 3:** Costs of EGFR testing, erlotinib and afatinib prescriptions

	**Costs to Medicare**	**Patient out-of-pocket costs**
**Cost of EGFR testing (*N* = 1086)**		
Mean (SD)	$1767 ($1511)	$350 ($339)
Median	$1,483	$293
Interquartile range	$798, $2234	$96, $477
Range	($0-$14,661)	($0-$3028)
**Average cost of a 30-day prescription of erlotinib (*N* = 394)**		
Mean (SD)	$5938 ($882)	$594 ($725)
Median	$6,114	$240
Interquartile range	$5460, $6615	$1, $973
Range	($2180-$7390)	($0-$2838)
**Average cost of a 30-day prescription of afatinib (*N* = 42)**		
Mean (SD)	$6267 ($735)	$605 ($722)
Median	$6,239	$471
Interquartile range	$5898, $6841	$1, $902
Range	($4203-$7256)	($0-$2771)

**Table 4:** Erlotinib and afatinib use within 6 months by EGFR testing

	**No EGFR test**	**EGFR test**	***p*-value**
**Number of erlotinib prescriptions among users**	*N* = 180	*N* = 214	0.846
Mean (SD)	4.5 (2.11)	4.6 (2.00)	
Median	5.0	5.0	
Interquartile range	3.0, 6.0	3.0, 6.0	
Range	(1.0–9.0)	(1.0–11.0)	
**Number of afatinib prescriptions among users**	*N* = 20	*N* = 22	0.959
Mean (SD)	3.9 (1.97)	4.0 (2.01)	
Median	4.5	4.0	
Interquartile range	2.0, 5.0	2.0, 6.0	
Range	(1.0–7.0)	(1.0–8.0)	

**Fig. 1:**
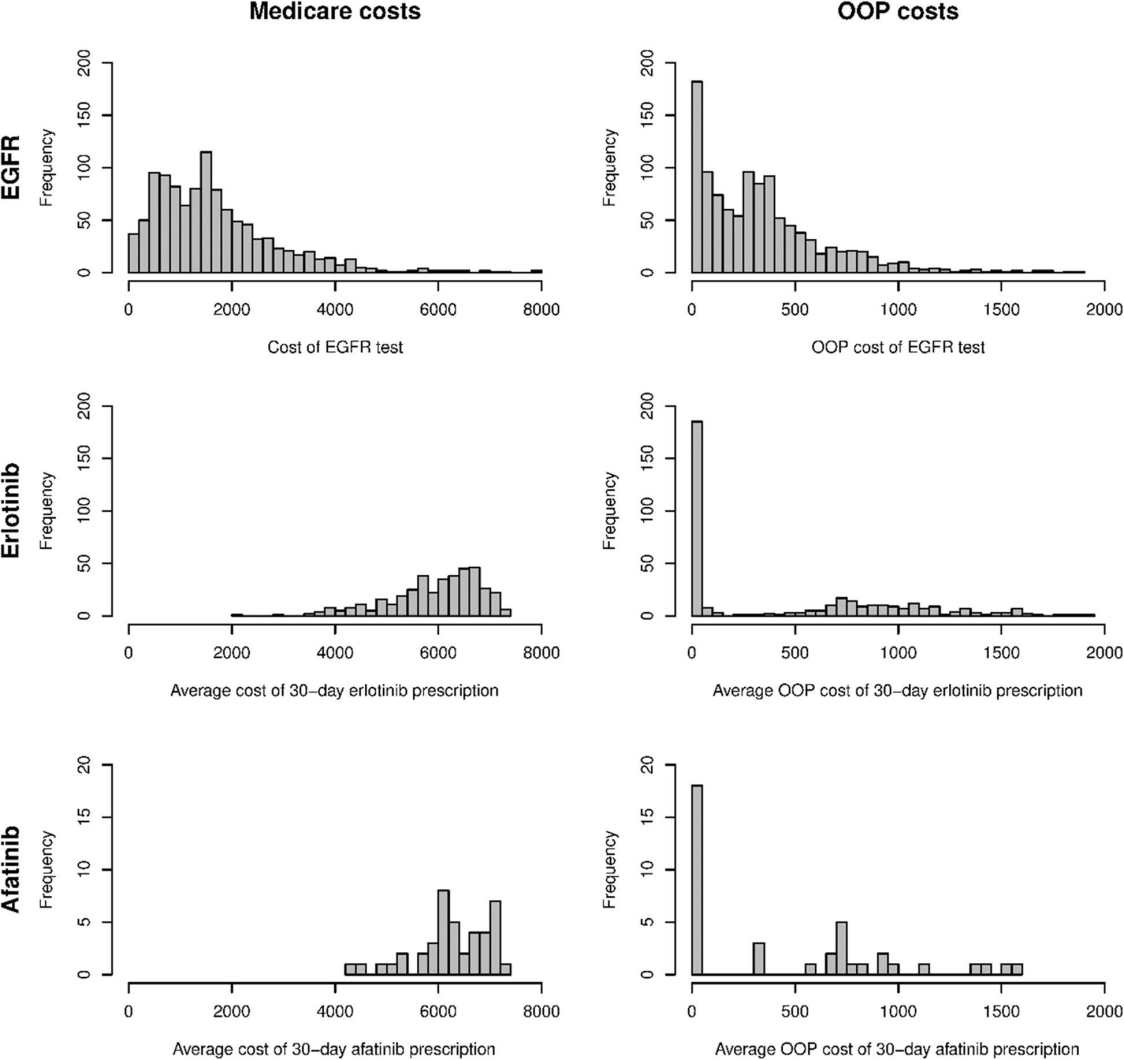
Histograms for Medicare costs (left column) and patient out-of-pocket costs (right column) for EGFR testing (top row) and EGFR targeted therapies erlotinib (middle row) and afatinib (bottom row). A small number of patients with Medicare costs for EGFR > $8000, and out-of-pocket costs > $2000 for EGFR, erlotinib, and afatinib are not shown in the figures. The total number of patients excluded from the figures is less than 50, while the exact number of patients excluded for each group is masked per SEER-Medicare user agreement for confidentiality

Costs for EGFR testing and EGFR targeted therapies exhibited large variation as demonstrated by the histograms in Fig. 1. Both Medicare and patient OOP costs for EGFR testing were markedly right-skewed, with most of the observations costing < $2,500 for Medicare costs and < $500 for patient OOP costs. The costs of EGFR targeted therapies were substantially higher. For Medicare, therapy costs were considerably left-skewed with most observations showing erlotinib and afatinib costing above $5,000 for 30 days of supply. The patient OOP costs were vastly different due to many patients having zero OOP costs while some had OOP costs > $1,000 per 30 days of supply.

A sensitivity analysis was performed including CPT codes for panel testing and laboratory developed test of cell free DNA. Including the CPT 81,445 and 81,455 (Genomic Sequencing Procedures and Other Molecular Multianalyte Assays) added 34 patients who might have potentially received EGFR through panel testing; while including CPT 81,479 (unlisted molecular pathology procedure) added 115 patients. Due to the overlapping of patients who received 81,445/81455 and 81,479, including all three additional CPT codes added 140 patients in total. In the scenario where patients who received these three additional codes all received EGFR testing, we could observe a moderate increase in EGFR testing rate from 46 to 52% in our study sample.

## Discussion

This study offers a population-based perspective on the rates and costs of EGFR testing among patients with newly diagnosed metastatic lung adenocarcinoma from January 2013 to December 2015 using the SEER-Medicare database. In our study, we observed that less than half (46%) of the 2,362 patients with newly diagnosed metastatic lung adenocarcinoma were tested for EGFR mutations within 6 months of diagnosis. When including CPT codes that could indicate EGFR testing conducted as part of a panel test, the rate of testing increased from 46 to 52%. This shows that there was clear underutilization of EGFR testing during the study time period.

We observed a significant increase in the use of EGFR testing over the study period. In 2013, 38% of patients were tested compared to 51% in 2014 and 49% in 2015. This suggests that recommendations by professional societies are positively correlated with improvement in the uptake of EGFR testing, as the guidelines recommending EGFR testing for all patients were released by the CAP/IASLC/AMP in July of 2013, and were endorsed by the ASCO in October of 2014 [7, 8]. The testing rate in our study was higher than two prior studies which also examined the EGFR testing rates in Medicare patients and concluded underutilization [18, 19]. However, these studies included data only up to 2013 and the patient inclusion criteria and time windows considered for EGFR testing were different. Another study in 2017 using MarketScan data on commercially insured patients from 2012 to 2014, reported an overall testing rate of 18%, and only 42% of patients who received erlotinib were tested [21]. However, that study did not incorporate histology data, and our study provides a more accurate and reliable estimate of the testing rate by limiting the study sample to patients with lung adenocarcinoma.

Notably, we found that many patients received targeted therapy without testing. More specifically, out of the patients who received erlotinib, 46% did not receive EGFR testing. Similarly, 48% of the patients treated with afatinib did not receive EGFR testing. EGFR targeted therapy is not recommended for patients that lack an EGFR sensitizing mutation. Multiple studies have demonstrated that EGFR targeted treatment can be less effective than standard chemotherapy in patients without sensitizing mutations [14–17, 31]. For example, the TORCH trial demonstrated significantly inferior overall survival for first-line erlotinib compared to first-line chemotherapy among unselected patients with advanced NSCLC [17]. Similarly, in the adjuvant setting, two trials showed no benefit of TKI in patients not selected based on EGFR mutation status [32, 33]. Therefore, failure to use molecular testing before treatment can result in the implementation of therapies that are not the most beneficial for the individual patient.

Importantly, our study addresses the paucity of knowledge of the costs of EGFR testing to Medicare and patient OOP cost, as well as EGFR targeted therapy prescriptions. We found that the cost of targeted therapy was substantially higher than the cost of EGFR testing. The median Medicare costs for 30 days of targeted therapy were above $6,000, while the testing costed less than $1,500. The cost comparison is even starker considering that the cost of testing is a one-time payment, while targeted therapy is a recurrent monthly payment. EGFR targeted therapy costs substantially more than traditional chemotherapy, and has been shown to have low efficacy in patients without a targetable mutation [14–17]. It is important, therefore, to ensure that testing is used to provide this treatment only to those patients who will benefit. Indeed, the high percentage of patients receiving targeted therapy without testing might be considered problematic. It highlights a missed opportunity to not only improve patient clinical outcomes but also reduce costs and conserve resources in the healthcare system.

Although Medicare carried most of the cost burden for both EGFR testing and targeted therapy prescriptions, these therapies still amount to a substantial and highly variable financial burden on patients with median OOP costs at $240 (IQR: $1, $973) and $471 (IQR: $1, $902) for 30-day prescription of erlotinib and afatinib respectively. Over the last several years the term “financial toxicity” has emerged to describe the negative financial impact of cancer treatment on patients. Financial toxicity related to cancer treatment, and specifically targeted therapies, has been shown to affect patient outcomes including quality of life and overall wellbeing [34–36]. In 2009, ASCO release a guidance statement recognizing the increasing cost of cancer treatment as an issue and highlighting the need for interventions [37]. Our study identifies low EGFR testing rates as a potential area of improvement that can help reduce the potentially unnecessary use of targeted therapy and therefore decrease the financial burden on patients without sensitizing mutations.

Although cost-effectiveness of EGFR targeted therapy is not the focus of this current study, it is noteworthy that there has been a growing literature on the cost-effectiveness of EGFR targeted compared to cytotoxic chemotherapy. These studies have shown mixed evidence on the cost-effectiveness of EGFR targeted therapy by country and by EGFR targeted agents [38–42]. More future studies are needed to further evaluate the cost-effectiveness of targeted therapies in different settings.

We also found significant disparities in EGFR testing based on urban/rural status, which is consistent with a prior study that also noted lower testing rates in rural areas [19]. There is an extensive body of work that highlights the disparities in cancer treatment and outcomes in rural and urban settings [43–45]. Our results suggest that these disparities extend to molecular testing and that efforts are needed to improve access to EGFR testing for rural patients.

This study has several limitations. There is a possibility that some patients lacked available or adequate tissue sampling that could be submitted for molecular testing and therefore did not receive EGFR mutation testing. The database used for this observational study only captures the use of testing but does not provide testing result. In addition, it does not include information on tissue availability, and patients that were tested as part of a research study might not have claims to Medicare and would therefore not be captured in our data. However, participation in research studies is unlikely to significantly impact our results, as the proportion of cancer patients above 65 years of age in the U.S. that participate in clinical trials is very low with enrollment fraction below 0.5% [46, 47]. Another limitation is that the database used in this study does not provide information on the line of therapy. Hence it is possible that some patients in the study cohort received targeted therapy as second- or third-line therapy. EGFR mutation testing was not required for second line treatment in the time period of our study [48]. Therefore the inclusion of patients who were not treated with first line EGFR targeted agents could lead to an artificially low testing rate. However, we limited the study cohort to newly diagnosed patients and examined only the first 6 months after diagnosis. The study is based on SEER-Medicare data focusing on Medicare patients above 65 years of age. Previous studies have suggested a correlation between age and the rate of EGFR mutation [49, 50]. It is important to note that because our study sample does not include patients younger than 65, our findings are not applicable to this population.

Molecular testing and targeted therapies for lung cancer have been evolving rapidly. Studies in the literature showed varying speed of practice changes in terms of adoption of new innovative therapies. For example, one study found that a majority of patients were treated with anti-PD1 agents just 4 months following US Food and Drug Administration approval [51]. Another study examining the adoption of immunotherapy in 123 practices treating 43,697 advanced NSCLC patients found significant variability in the adoption of new therapy including some rapid adopters, some slower adopters with limited adoption even after 2 years, some later adopters accelerating after 18 months, and some decelerating adopters slowing markedly after 1 year [52]. Therefore, our data may not be representative of current clinical practices, and may only reflect the practice pattern shortly after the guideline change. Additionally, a growing number of providers are offering next-generation sequencing profiling, and many cancer centers also have liquid molecular profiling available in addition to tissue profiling or genotyping. Future studies are warranted to examine the more recent trends in the use and costs of comprehensive molecular profiling and targeted therapies. Lastly, patients with HMO coverage were excluded from the analysis as claims data is not available for this population. HMO patients represent over 50% of Medicare beneficiaries in some markets, and their exclusion limits the generalizability of our results [53].

To the best of our knowledge, this is the first large population-based study on both the costs and rates of EGFR testing among Medicare patients with metastatic lung adenocarcinoma. We analyzed data after 2013, which reflect the result of changes in guidelines and recommendations from CAP/IASLC/AMP and ASCO. We found that, although there is still substantial underutilization rates of EGFR testing, the rates did improve after guidelines recommended universal testing for all patients with metastatic lung adenocarcinoma who were being considered for first line targeted therapy. We also found significant disparities in EGFR testing rates in rural areas compared to larger metropolitan areas suggesting the need for interventions to improve the uptake of molecular testing in rural areas. Further, our study showed that the cost of EGFR testing is substantially less than the cost of targeted therapies, and close to half of the patients receiving targeted therapies did not undergo the relevant testing. Such findings highlight the need to improve adherence to testing guidelines, which can yield a three-fold benefit of improving patient outcomes, reducing cost burdens on Medicare, and mitigating the financial toxicity for metastatic lung adenocarcinoma patients.

## Supplementary Information

**Additional file 1:** **Appendix** **Figure 1.** Studycohort inclusion and exclusion criteria. STROBEStatement—Checklist of items that should be included in reports of cohort studies.

## Data Availability

The data generated or analyzed during this study are included in this published article are available at: https://healthcaredelivery.cancer.gov/seermedicare/. Data requests will be reviewed by SEER-Medicare and will need to comply with SEER-Medicare data use agreement [54].
